# Supplementary material for: Health Beliefs and Socioeconomic Determinants of COVID-19 Booster Vaccine Acceptance: An Indonesian Cross-Sectional Study
Source: Vaccines (Basel). 2022 May 5;10(5):724. doi: 10.3390/vaccines10050724 (PMC9146460; doi:10.3390/vaccines10050724)
Supplement: Supplementary file 1 [file vaccines-10-00724-s001.zip › Table S1.pdf]

Table S1. Item list for health belief measures.

Variables	Cronbach α
**Perceived Threat**<br>• It is likely that I will get ill from COVID-19<br>• COVID-19 could possibly affect my health<br>• I believe I am seriously at risk of getting ill from COVID-19<br>• COVID-19 is harmful to my health<br>• COVID-19 can severely affect my health<br>• The health effects of COVID-19 infection are of serious concern<br>• COVID-19 infection may cause me long-term health issues<br>• COVID-19 infection may lead to my death	0.922
**Perceived Barriers**<br>• It's going to be difficult to get information regarding COVID-19 vaccine booster<br>• It's going to be difficult to get information on where to get COVID-19 vaccine booster<br>• It's going to be difficult to get scheduled for COVID-19 vaccine booster<br>• It's going to be difficult for me to get transportation to reach the COVID-19 vaccine booster service locations<br>• The COVID-19 vaccine booster service location would be too far from me<br>• Healthcare providers would not help me to get COVID-19 vaccine booster	0.948
**Perceived Harms**<br>• COVID-19 vaccine boosters may cause serious short-term adverse events<br>• COVID-19 vaccine boosters may cause long-term health issues<br>• COVID-19 vaccine boosters may lead to long-term disabilities<br>• COVID-19 vaccine boosters may lead to death<br>• Boosters -related adverse events may disturb my work and employment	0.926
**Perceived Benefits**<br>• Booster doses will significantly reduce risk of COVID-19 infection<br>• Booster doses will significantly reduce risk of transmitting COVID-19 to others<br>• Booster doses will significantly reduce risk of hospitalization with COVID-19<br>• Booster doses will significantly reduce risk of long-term health issues caused by COVID-19<br>• Booster doses will significantly reduce risk of death from COVID-19	0.945
