# Supplementary material for: Health Beliefs and Socioeconomic Determinants of COVID-19 Booster Vaccine Acceptance: An Indonesian Cross-Sectional Study
Source: Vaccines (Basel). 2022 May 5;10(5):724. doi: 10.3390/vaccines10050724 (PMC9146460; doi:10.3390/vaccines10050724)
Supplement: Supplementary file 1 [file vaccines-10-00724-s001.zip › Table S2.pdf]

Table S2. Stratified multinomial logistic regression model for COVID-19 vaccine booster acceptance based on respondent's location.

	**Jakarta (n = 1,894)**		**Bali (n = 780)**	
**Variables**	Planned to Accept aOR (95% CI)	Already Accepted aOR (95% CI)	Planned to Accept aOR (95% CI)	Already Accepted aOR (95% CI)
**Health Belief**				
**Perceived threat**				
**Low**	1	1	1	1
**High**	1.72 (1.35 – 2.18)**	2.56 (1.69 – 3.88)**	1.53 (1.00 – 2.35)	2.02 (1.25 – 3.25)**
**Perceived barriers**				
**Low**	1	1	1	1
**High**	0.71 (0.55 – 0.91)**	0.28 (0.18 – 0.44)**	0.49 (0.31 – 0.78)**	0.29 (0.17 – 048)**
**Perceived harms**				
**Low**	1	1	1	1
**High**	0.43 (0.33 – 0.55)**	0.46 (0.29 – 0.72)**	0.63 (0.39 – 1.03)	0.51 (0.30 – 0.87)*
**Perceived benefits**				
**Low**	1	1	1	1
**High**	2.85 (2.22 – 3.66)**	1.99 (1.27 – 3.10)	2.66 (1.69 – 4.20)**	1.59 (0.95 – 2.64)
**Media Influence and Trust**				
**Influence of print media**				
**Low**	1	1	1	1
**High**	1.71 (1.29 – 2.26)**	1.23 (0.77 – 1.97)	1.06 (0.63 – 1.77)	1.25 (0.71 – 2.19)
**Influence of television**				
**Low**	1	1	1	1
**High**	1.11 (0.79 – 1.56)	0.43 (0.24 – 0.77)**	1.37 (0.78 – 2.41)	1.03 (0.55 – 1.94)
**Influence of radio**				
**Low**	1	1	1	1
**High**	0.99 (0.76 – 1.31)	1.20 (0.76 – 1.90)	0.78 (0.48 – 1.27)	0.84 (0.49 – 1.43)
**Influence of online media**				

**Low**	1	1	1	1
**High**	0.89 (0.61 – 1.29)	1.20 (0.65 – 2.23)	1.08 (0.55 – 2.13)	0.79 (0.37 – 1.67)
**Influence of social media**				
**Low**	1	1	1	1
**High**	1.96 (1.41 – 2.73)**	1.75 (1.02 – 3.01)*	1.16 (0.65 – 2.07)	1.44 (0.76 – 2.73)
**Trust in authoritative sources**				
**Low**	1	1	1	1
**High**	1.33 (1.02 – 1.73)*	1.18 (0.76 – 1.84)	1.88 (1.19 – 2.98)**	1.38 (0.82 – 2.33)
**Demographics and COVID-19 History**				
**Sex**				
**Male**	1	1	1	1
**Female**	1.18 (0.89 – 1.56)	0.81 (0.53 – 1.26)	1.15 (0.73 – 1.80)	1.17 (0.72 – 1.92)
**Age (per incremental years)**	1.00 (0.99 – 1.01)	1.02 (1.00 – 1.05)	1.01 (0.99 – 1.04)	1.03 (1.01 – 1.06)*
**Religion**				
**Islam**	1	1	1	1
**Non-Islam**	2.99 (1.92 – 4.66)**	3.28 (1.88 – 6.39)**	1.57 (0.99 – 2.48)	2.91 (1.68 – 5.05)**
**Education**				
**Not completed high school**	0.92 (0.63 – 1.34)	0.46 (0.13 – 1.59)	0.27 (0.09 – 0.80)*	1.00 (0.30 – 3.39)
**Completed high school**	1	1	1	1
**Completed college**	1.38 (1.03 – 1.86)*	3.95 (2.44 – 6.39)**	1.33 (0.81 – 2.17)	2.50 (1.40 – 4.48)**
**Employment**				
**Full-time employment**	1	1	1	1
**Part-time employment**	0.88 (0.62 – 1.25)	0.44 (0.25 – 0.79)**	0.61 (0.32 – 1.15)	0.45 (0.23 – 0.88)*
**Student**	0.71 (0.42 – 1.20)	0.57 (0.22 – 1.48)	0.74 (0.33 – 1.66)	0.27 (0.10 – 0.68)**
**Stay-at-home wife**	0.76 (0.52 – 1.12)	0.16 (0.07 – 0.37)**	0.72 (0.32 – 1.60)	0.11 (0.04 – 0.32)**
**Unemployed**	0.70 (0.46 – 1.08)	0.20 (0.08 (0.49)**	0.47 (0.22 – 0.99)*	0.19 (0.08 – 0.44)**
**Monthly income**				
**< IDR 1 million**	1	1	1	1

**IDR 1 million – IDR 3 million**	1.10 (0.80 – 1.52)	0.98 (0.46 – 2.09)	0.91 (0.50 – 1.65)	0.56 (0.28 – 1.15)
**IDR 3 million – IDR 5 million**	0.96 (0.68 – 1.37)	0.85 (0.39 – 1.86)	1.11 (0.53 – 2.32)	0.65 (0.27 – 1.52)
**IDR 5 million**	1.59 (1.02 – 2.46)**	1.52 (0.66 – 3.50)	1.41 (0.55 – 3.61)	1.09 (0.39 – 3.01)
**Health insurance**				
**Subsidized public insurance**	1	1	1	1
**Unsubsidized public insurance**	1.09 (0.82 – 1.44)	1.41 (0.84 – 2.36)	1.30 (0.75 – 2.27)	3.58 (1.83 – 7.02)**
**Private insurance**	0.99 (0.74 – 1.31)	1.12 (0.65 – 1.93)	0.60 (0.34 – 1.04)	1.08 (0.54 – 2.17)
**COVID-19 infection history**				
**Never infected**	1	1	1	1
**Infected, never hospitalized**	1.16 (0.79 – 1.73)	0.65 (0.35 – 1.23)	1.53 (0.66 – 3.53)	0.23 (0.05 – 1.23)
**Infected and hospitalized**	0.83 (0.39 – 1.78)	0.74 (0.24 – 2.25)	0.32 (0.07 – 1.44)	1.04 (0.42 – 2.59)
**COVID-19 infection in family/friends**				
**No infection**	1	1	1	1
**Infection only**	1.18 (0.84 – 1.65)	1.18 (0.68 – 2.07)	1.15 (0.64 – 2.08)	1.43 (0.76 – 2.67)
**Hospitalization**	0.99 (0.65 – 1.51)	1.30 (0.67 – 2.51)	1.23 (0.66 – 2.29)	0.95 (0.46 – 1.95)
**Mortality**	1.17 (0.84 – 1.63)	1.56 (0.94 – 2.60)	1.50 (0.78 – 2.89)	1.51 (0.72 – 3.15)

*$p < 0.05$; **$p < 0.01$
